# Development of methodology for assessing steroid-tapering in clinical trials for biologics in asthma

**DOI:** 10.1186/s12931-022-01959-1

**Published:** 2022-03-04

**Authors:** Stephanie Korn, Peter Howarth, Steven G. Smith, Robert G. Price, Steven W. Yancey, Charlene M. Prazma, Elisabeth H. Bel

**Affiliations:** 1 Pulmonary Department, Universitätsmedizin Mainz, Mainz, Langenbeckstr Germany; 2 Global Medical Franchise, GSK House, Brentford, Middlesex UK; 3 Respiratory Therapeutic Area, GSK, Research Triangle Park, NC USA; 4 Biostatistics, GSK, Stevenage, Hertfordshire UK; 5 Global Respiratory Franchise, GSK, Research Triangle Park, NC USA; 6 Department of Respiratory Medicine, Amsterdam UMC, Location AMC, University of Amsterdam, Amsterdam, The Netherlands; 7 Clinical Research Centre Respiratory Disease, IKF Pneumologie GmbH & Co. KG, Haifa-Allee 24, 55128 Mainz, Germany; 8 Thoraxklinik Heidelberg, Röntgenstr. 1, 69126 Heidelberg, Germany

**Keywords:** Asthma, Biologics, Efficacy, Methodology, OCS reduction, OCS-sparing, OCS-tapering, Patient selection, Treatment response

## Abstract

**Background:**

Long-term use of oral corticosteroids (OCS) is associated with a risk of adverse events and comorbidities. As such, a goal in assessing the efficacy of biologics in severe asthma is often to monitor reduction in OCS usage. Importantly, however, OCS dose reductions must be conducted without loss of disease control.

**Main body:**

Herein, we describe the development of OCS-sparing study methodologies for biologic therapies in patients with asthma. In particular, we focus on four randomized, placebo-controlled, parallel-group studies of varying sizes (key single-center study [n = 20], SIRIUS [n = 135], ZONDA [n = 220], VENTURE [n = 210]) and one open-label study (PONENTE [n = 598]), which assessed the effect of asthma biologics (mepolizumab, benralizumab or dupilumab) on OCS use using predefined OCS-tapering schedules. In particular, we discuss the evolution of study design elements in these studies, including patient eligibility criteria, the use of tailored OCS dose reduction schedules, monitoring of outcomes, the use of biomarkers and use of repetitive assessments of adrenal function during OCS tapering.

**Conclusion:**

Taken together, these developments have improved OCS-sparing asthma studies in recent years and the lessons learned may help with optimization of further OCS-sparing studies, and potentially clinical practice in the future.

**Supplementary Information:**

The online version contains supplementary material available at 10.1186/s12931-022-01959-1.

## Background

Patients with severe asthma often require regular oral corticosteroids (OCS) to maintain asthma control [1, 2]. For patients with severe allergic or severe eosinophilic asthma who experience poor symptom control and/or frequent asthma exacerbations, despite the use of high-dose inhaled corticosteroids (ICS), long-acting β_2_-agonists (LABA) and/or maintenance OCS, the use of biologic treatments as add-on therapy is becoming the new standard of care [2]. Five biologics are now approved for the treatment of severe asthma. Omalizumab is an anti-immunoglobulin E antibody approved for the treatment of moderate-to-severe persistent allergic asthma [3, 4]. Mepolizumab, reslizumab and benralizumab are anti–interleukin-5/anti–interleukin-5 receptor humanized monoclonal antibodies approved in the USA and Europe for the treatment of severe eosinophilic asthma [5–10]. Most recently, dupilumab, an anti–interleukin-4 receptor alpha monoclonal antibody was approved in the USA for the treatment of moderate-to-severe eosinophilic or OCS-dependent asthma and in Europe for the treatment of severe asthma with type 2 inflammation [11, 12].

Studies assessing the efficacy of biologics for the treatment of asthma often aim to reduce the use of OCS, in turn, reducing the risk of adverse events (AEs) and comorbidities associated with chronic OCS use [13–15]. However, this must be done without loss of disease control; current guidance and data from less recent publications on the strategy for reducing maintenance OCS use in patients initiating biologic treatment are insufficient [2, 16, 17]. In a clinical trial setting, suitable patient selection criteria and effective OCS dose tapering strategies are key when assessing biologic asthma therapies, to ensure trial outcomes are clinically informative [18].

Here, we describe the development of OCS-sparing study methodologies for biologic therapies in patients with asthma. In particular, we focus on lessons learned from four randomized, placebo-controlled, parallel-group studies: a key single-center study (mepolizumab) [19], SIRIUS (mepolizumab) [20], ZONDA (benralizumab) [21], VENTURE (dupilumab) [22] and one open-label study (PONENTE [benralizumab]) [23, 24]. All these studies assessed the effect of asthma biologics on OCS use using predefined OCS-tapering schedules (Table 1).

**Table 1:** Asthma biologic studies with pre-defined OCS-tapering schedules

Study	Study initiation	Trial type	Treatment	Dosing frequency	Patients (ITT)
Key single-center study [19] (NCT00292877)	2005	Phase 2 RCT Double-blind Parallel-group	Mepolizumab 750 mg IV or placebo	Every 4 weeks for 16 weeks	20
SIRIUS [20] (NCT01691508)	2012	Phase 3 Multicenter RCT Double-blind Parallel-group	Mepolizumab 100 mg SC or placebo	Every 4 weeks for 24 weeks	135
ZONDA [21] (NCT02075255)	2014	Phase 3 Multicenter RCT Double-blind Parallel-group	Benralizumab 30 mg SC or placebo	Every 4 weeks for 12 weeks followed by every 4 weeks or every 8 weeks for 16 weeks (total duration: 28 weeks)	220
VENTURE [22] (NCT02528214)	2015	Phase 3 Multicenter RCT Double-blind Parallel-group	Dupilumab 300 mg SC or placebo	Every 2 weeks for 24 weeks	210
PONENTE [23, 24] (NCT03557307)	2018	Phase 3b Multicenter Open-label Single-arm	Benralizumab 30 mg SC	Every 4 weeks for 8 weeks (first 3 doses) followed by every 8 weeks until end of treatment*	598

*ITT* intent-to-treat population, *IV* intravenous, *OCS* oral corticosteroid, *RCT* randomized controlled trial, *SC* subcutaneous

*The open-label benralizumab treatment period consists of a 4-week induction phase, a variable OCS tapering phase and a 24–32-week maintenance phase

### Patient selection

Across the OCS-tapering studies, patients had similar asthma disease phenotypes (Table 2); all studies targeted patients with severe asthma who were on regular maintenance OCS [19–24]. Continuous OCS use was required for all studies but the amount of time patients were required to have been receiving maintenance OCS differed (Table 2) [19–24]. There were also differences in the prednisone equivalent OCS dose required for eligibility for the five studies (Table 2) [19–24]. Additionally, patients in SIRIUS were stratified at randomization by history of maintenance OCS use (< 5 years versus ≥ 5 years) based on an assumption that OCS tapering would be more difficult in patients who had been receiving maintenance OCS for a long period [20]. Further differences in eligibility criteria included varying exacerbation history and blood eosinophil count requirements (Table 2).

**Table 2:** Eligibility criteria for OCS-sparing studies

Study reference	Patient age (years)	Asthma phenotype	Receiving OCS at enrollment	Duration of continuous OCS use	OCS dose* range at baseline (mg/day)	Asthma diagnosis criteria^†^
Key single-center study [19] (NCT00292877)	18–70	Asthma with persistent sputum eosinophilia despite OCS	Yes	≥ 4 weeks	5–25	• Variable airway obstruction^‡^ in the previous 8 years • Sputum eosinophil > 3%
SIRIUS [20] (NCT01691508)	≥ 12	Severe eosinophilic asthma	Yes	≥ 6 months Stable: for ≥ 1 month	5–35	• Peripheral blood eosinophil count ≥ 300 cells/µL in the 12 months prior to screening or ≥ 150 cells/µL during OCS dose optimization period • Airway obstruction^§^, reversibility^║,¶^, hyperresponsiveness** within 12 months or variability during OCS dose optimization period^††^ • Very high-dose ICS^‡‡^ plus ≥ 1 controller for ≥ 3 months
ZONDA [21] (NCT02075255)	18–75	Severe eosinophilic asthma	Yes	≥ 6 months	7.5–40	• Peripheral blood eosinophil count ≥ 150 cells/µL at enrollment • ≥ 1 exacerbation in the prior 12 months • Medium-to-high dose ICS^§§^ for ≥ 12 months • LABA for ≥ 12 months
VENTURE [22] (NCT02528214)	≥ 12	OCS dependent severe asthma^║║^	Yes	≥ 6 months Stable: for ≥ 1 month	5–35	• No eosinophil count requirement • High-dose ICS^¶¶^ (stable: for ≥ 1 month) plus ≥ 1 controller for ≥ 3 months • Airway obstruction^§^, reversibility^║^ or hyperresponsiveness** within 12 months
PONENTE [23, 24] (NCT03557307)	≥ 18	Severe eosinophilic asthma	Yes	≥ 3 months Stable: for ≥ 4 weeks	≥ 5	• Peripheral blood eosinophil count ≥ 150 cells/µL at enrollment or ≥ 300 cells/µL in the 12 months prior to enrollment • High-dose ICS^¶¶^ plus LABA for ≥ 6 months

*FEV*_*1*_ forced expiratory volume in 1 s, *FVC* forced vital capacity, *ICS* inhaled corticosteroid, *LABA* long-acting β_2_-agonist, *OCS* oral corticosteroid, *PC20* provocative concentration of methacholine resulting in a 20% decrease in FEV_1_, *PD20* provocative dose of methacholine resulting in a 20% decrease in FEV_1_

*Prednisone or equivalent; ^†^prior to enrollment, unless otherwise stated; ^‡^at least a 25% reduction in FEV_1_ at the time of exacerbation; ^§^pre-bronchodilator FEV_1_ < 80% predicted in patients ≥ 18 years of age (in SIRIUS, patients 12–17 years of age had to have pre-bronchodilator FEV_1_ < 90% predicted or FEV_1_/FVC ratio < 0.8; In VENTURE, adolescents had to have pre-bronchodilator FEV_1_ ≤ 90% predicted); ^║^FEV_1_ ≥ 12% and 200 mL; ^¶^or FEV_1_ ≥ 20% between two consecutive clinical visits (excluding exacerbations); **PC20 < 8 mg/mL or PD20 < 7.8 µmol; ^††^ > 20% diurnal variability in peak flow for ≥ 3 days during OCS dose optimization; ^‡‡^ ≥ 880 µg/day fluticasone propionate (12–17 years of age ≥ 440 µg/day); ^§§^ > 250 μg fluticasone dry powder formulation equivalents total daily dose; ^║║^based on the Global Initiative for Asthma (GINA) 2014 guidelines (a history of respiratory symptoms such as wheeze, shortness of breath, chest tightness and cough that vary over time and in intensity, together with variable expiratory airflow limitation); ^¶¶^fluticasone propionate at a total daily dose of > 500 μg or equipotent equivalent

The evolution of eligibility criteria since the key single-center study has led over time to study populations in the larger multicenter studies that more precisely reflect patients for whom biologic therapy is appropriate in real-world clinical practice. The inclusion criteria for the most recent studies ensured that patients have severe asthma with active eosinophilic inflammation. In addition, the need for stable OCS use prior to initiation of a biologic has been introduced, with three of the four most recent studies (SIRIUS, VENTURE and PONENTE) requiring a stable OCS dose prior to enrollment/screening.

### Trial design and OCS-tapering schedule

In the key single-center study, the OCS dose was reduced providing the patient had not experienced a defined exacerbation and target doses were 0, 2.5 and 5 mg/day in patients requiring daily OCS doses of < 10, 10– < 15 and ≥ 15 mg/day at baseline, respectively [19]. Additional information detailing the criteria for not further reducing OCS dose during the OCS reduction period for each study can be found in the Additional file 1.

**Fig. 1:**
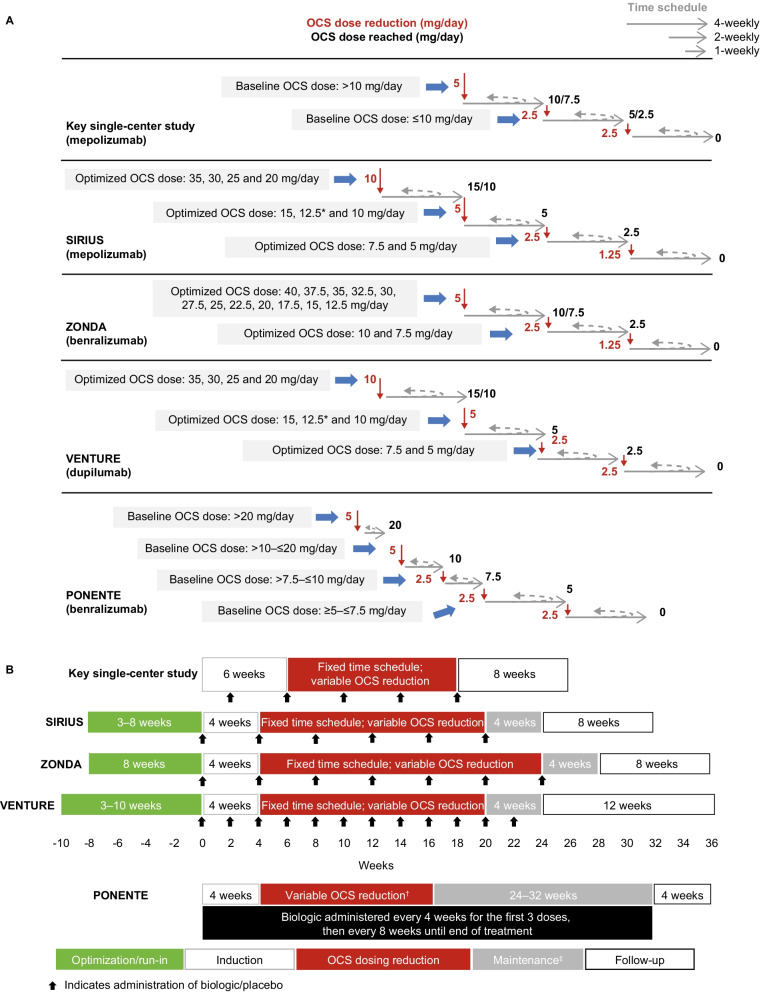
OCS-sparing schedules during the OCS dosing reduction period (**A**) and overall trial design (**B**). In the key single-center study, patients with a baseline OCS dose ≥ 15 mg/day were not permitted to reduce their dose below 5 mg/day and patients with baseline 10– < 15 mg/day were not permitted to reduce their dose below 2.5 mg/day; only patients with a baseline dose < 10 mg/day were permitted to reduce to 0 mg/day. In SIRIUS, patients with an optimized OCS dose of ≥ 25 mg/day were not permitted to reduce their dose to 0 mg/day (not permitted below 2.5 mg/day). In ZONDA, patients with an optimized dose of between 20 and 30 mg/day were not permitted to reduce their dose below 5 mg/day and patients with an optimized dose of 17.5 or 15 mg/day were held temporarily at 5 mg/day for at least 8 weeks before continuing OCS dose reductions; only patients who were receiving an optimized dose of ≤ 12.5 mg/day could reduce their dose to 0 mg/day. In VENTURE, patients with an optimized OCS dose of ≥ 35 mg/day were not permitted to reduce their dose below 2.5 mg/day; only patients receiving an optimized dose < 35 mg/day could reduce their dose to 0 mg/day. Further information detailing the criteria for not reducing OCS dose for each study can be found in the Additional file 1. Patients in ZONDA with documented failures of OCS dose reduction within 6 months prior to enrollment were not required to proceed through the OCS dose optimization phase (Additional file 1). *Starting doses of 12.5 mg/day had an initial reduction of 2.5 mg/day (to 10 mg/day) followed by a reduction of 5 mg/day (to 5 mg/day); ^†^variable duration with no minimum or maximum limits, depending on individual baseline OCS dose; ^‡^no further changes in OCS dose were permitted during this period. *OCS* oral corticosteroid

Building upon the OCS-tapering methodology used in the single-center study, the subsequent trials were able to use a more tailored approach (Fig. 1a and b). SIRIUS was the first study to include an OCS dose optimization phase, which aimed to reduce a patient’s OCS dose to the lowest possible effective dose while maintaining asthma control prior to randomization to biologic treatment [20]. During the 3–8-week OCS dose optimization phase in SIRIUS, OCS dose was reduced weekly until asthma worsening was observed, defined as worsening of asthma symptoms (a ≥ 0.5-point increase in Asthma Control Questionnaire [ACQ]-5 score) or an exacerbation. The optimized OCS dose was the dose that was one titration step higher than the OCS dose received when the patient saw an onset of asthma worsening; the patient then maintained this optimized dose for 2 weeks. Subsequently, patients entered the induction phase (4 weeks) where they were randomized to biologic or placebo treatment, stratified by previous duration of OCS use (< 5 years versus ≥ 5 years) as previously noted and received the optimized dose of OCS [20]. Following the induction phase, patients entered a 16-week dose reduction phase in which the OCS dose was reduced in a stepwise manner based on asthma control and symptoms of adrenal insufficiency, with a target of 0 mg/day only for those who had reached an optimized dose of < 25 mg/day prior to randomization (Fig. 1a) [20]. For those on an optimized dose ≥ 25 mg/day, a target of 0 mg/day was not possible within the 24-week study in order to protect the patient from the risk of adrenal crisis. It is unclear whether the inclusion of multiple sites in multiple countries impacted the optimization of OCS dose, given the likelihood that OCS management practices varied across these sites/countries, and this may also be applicable to the other multicenter studies reported in this article.

The subsequent ZONDA and VENTURE studies also included OCS dose optimization phases (8 weeks and 3–10 weeks, respectively, Fig. 1b) [21, 22]. The ZONDA study did not require a stable OCS dose at screening/enrollment; however, the run-in (OCS dose optimization) period included stabilization of OCS dose. Only patients who were receiving a dose of ≤ 12.5 mg/day at the end of the OCS dose optimization/run-in phase were eligible for a 100% dose reduction to 0 mg/day during the 20-week dose reduction phase (Fig. 1a) [21]. Patients in ZONDA with documented failures of OCS dose reduction within 6 months prior to enrollment were not required to proceed through the OCS dose optimization phase (Additional file 1). In VENTURE, the 16-week OCS dose reduction phase had a target dose of 2.5 mg/day and 0 mg/day for patients with an optimized OCS dose of ≥ 35 mg/day and < 35 mg/day, respectively (Fig. 1a) [22]. The lowest effective OCS dose was defined by the emergence of asthma symptoms (a ≥ 0.5-point increase in ACQ-5 score), the occurrence of an exacerbation or any clinically significant event leading to an increase in dose.

In the PONENTE study, no OCS dose optimization phase was included, likely due to the open-label design; however, the OCS dose reduction phase had an initial target of 5 mg/day, with further dose reductions of 2.5 mg/day permitted for patients with no adrenal insufficiency, assessed by adrenocorticotropic hormone stimulation testing (Fig. 1a) [24]. This was the first study to formally introduce adrenal function testing as part of the OCS reduction protocol. For patients with partial adrenal insufficiency or symptoms suggestive of adrenal insufficiency (in the absence of abnormal adrenal insufficiency tests), OCS dose was only reduced by 1 mg/day every 4 weeks once a dose of 5 mg/day was reached. However, in case of complete adrenal insufficiency, no further tapering in the OCS dosage was allowed.

The modifications in study design since the key single-center study (n = 20) was published in 2009 [19], have focused on refinement of the OCS dose optimization strategy, which has implications for the study outcomes. An insufficient optimization period may result in a greater reduction in OCS dose while on study treatment, which has the potential to overemphasize the active treatment effect when taken in isolation of placebo response. Furthermore, the longer the tapering period, the greater the potential to demonstrate a larger reduction in dose, so this aspect of study design may affect outcomes reported during the study. It is also worth noting that the use of tailored OCS dose reductions based on baseline optimal OCS dose ensures a balance between minimizing OCS dose, maintaining asthma control and minimizing any impact of adrenal insufficiency on patients.

### Assessing asthma control during OCS-tapering

During tapering, OCS dose is reduced based on the level of asthma control, as well as symptoms of adrenal sufficiency (as monitored in SIRIUS, VENTURE and PONENTE). ACQ score, forced expiratory volume in 1 s (FEV_1_) measurements, morning peak expiratory flow (PEF) measurements, occurrence/history of exacerbations, in addition to blood eosinophil counts, feature as indicators of asthma control across the five OCS tapering studies (see also Additional file 1) [19–24]. Additionally, patients in SIRIUS used eDiaries to report their daily symptoms allowing close monitoring of asthma control by study investigators [20]. The use of eDiaries has now become commonplace in subsequent studies [21–23]. As such, methods to monitor asthma symptom control have broadened, from the sole reliance on clinical monitoring to a combination of clinical monitoring and patient-reported monitoring using standardized questionnaires [19–23]. Such monitoring tools provide a much more holistic picture of asthma control throughout clinical studies.

### Biomarkers for effective OCS dose reduction

The use of biomarkers to monitor both the efficacy and safety of reducing OCS dose in these trials can support an individualized patient-focused approach. The OCS-sparing studies described here did not formally identify biomarkers to guide tapering, although serum cortisol concentration was used in the PONENTE study [23, 24].

Blood eosinophil counts may also serve as useful indicators for effective OCS-tapering in patients with eosinophilic inflammation, as patients with OCS-dependent asthma may present with elevated levels of type 2 inflammatory markers [25]. OCS-sparing studies have correlated elevated blood eosinophil counts to a loss of asthma disease control [19–22]. Furthermore, an inverse association between OCS dose and blood eosinophil count has been demonstrated in patients with severe eosinophilic asthma not treated with an asthma biologic, with reduced OCS dose being associated with increased eosinophil count [26, 27]. Moreover, a post hoc analysis of data from SIRIUS showed that patients with the lowest blood eosinophil counts at baseline (< 150 cells/µL) had the highest mean OCS dose at the end of the optimization phase [28]. As such, timely blood eosinophil count might be useful as a potential biomarker for effective OCS dose reduction prior to biologic treatment initiation, either during OCS dose optimization in a trial setting or in real-world clinical practice.

It is clear that long-term OCS use is associated with adrenal insufficiency [29]; therefore, symptomatic measurements for adrenal insufficiency (fatigue, lassitude, weakness, nausea and vomiting, and hypotension) were used during OCS-tapering in SIRIUS to determine the appropriateness of reducing OCS dose. This methodology has been developed further in the PONENTE study by evaluating hypothalamic–pituitary–adrenal (HPA) axis integrity for adrenal insufficiency as part of the OCS tapering protocol. Serum cortisol concentration was used as a biomarker for adrenal insufficiency, and an adrenocorticotropic hormone stimulation test was performed when morning cortisol concentrations were less than the normal range but higher than that anticipated for those with complete adrenal insufficiency [23, 24]. In a recent Delphi consensus statement on OCS tapering in asthma, consensus was reached on the need for physicians to assess for adrenal insufficiency, which should involve an endocrinologist or multidisciplinary approach [30]. Experts agreed that adrenal insufficiency should be assessed using fasting morning cortisol, with the use of a (short) tetracosactide/cosyntropin (e.g., Synacthen®) test in patients with intermediate results.

### Study outcomes

The need for OCS dose optimization and dose reduction phases of an appropriate length and design may be demonstrated by comparison of outcome results from OCS tapering studies. In VENTURE, the OCS dose was reduced by a median of 50% in the placebo-treated group, which is greater than that seen in the ZONDA and SIRIUS studies, (25% and 0% reduction with placebo, respectively)., This high placebo response in VENTURE is suggestive of potentially inadequate OCS optimization before randomization. When comparing treatment effects versus placebo, the proportion of patients with a ≥ 90% reduction in OCS dose was greater with biologic therapy versus placebo in each of the VENTURE, ZONDA and SIRIUS studies (24% difference between placebo and dupilumab in VENTURE, a 21–25% difference between placebo and benralizumab in ZONDA, and 12% difference between placebo and mepolizumab in SIRIUS). In the PONENTE study, most patients (63%) eliminated the use of OCS following treatment with benralizumab [24]. These study outcomes are not, however, comparable as there were differences between the studies in the length of the OCS dose optimization and OCS reduction phases. It is likely that differing study populations and varying OCS management practices also influenced study outcomes, particularly the differences in eosinophil count requirement prior to the study, severity of disease and duration of OCS therapy, given that the use of OCS in patients with asthma has become more judicious over time.

Of interest, although patients from SIRIUS, VENTURE, ZONDA and PONENTE all had uncontrolled asthma according to mean ACQ-5/6 scores, those in SIRIUS appeared to have the most severe asthma, demonstrated by the highest daily OCS dose prior to optimization (12.5–15 mg/day median dose for SIRIUS compared with 10.0 mg/day median dose in ZONDA and PONENTE and 11.8 mg/day mean dose in VENTURE). Moreover, 48% of patients in SIRIUS had been receiving OCS for ≥ 5 years, whereas the mean time since first OCS prescription in VENTURE was 1.7 years and 23% of patients in PONENTE had been taking OCS for < 1 year. Higher disease severity in SIRIUS was also indicated by the mean number of exacerbations experienced by each patient in the 12 months prior to the study; in SIRIUS, patients had experienced a mean of 2.9–3.3 severe exacerbations each in the 12 months prior to enrollment, compared with a mean of 2.0–2.2 per patient in VENTURE, while patients in ZONDA had experienced a mean of 2.5–3.1 exacerbations and those in PONENTE had experienced a mean of 3.0 exacerbations. These differences in baseline disease severity are reflected in the annualized exacerbation rate in the placebo group, which was highest in SIRIUS (2.1) compared with ZONDA (1.8) and VENTURE (1.6). Notably, the exacerbation definitions differed slightly between these studies in that exacerbations defined by OCS use required an increase of ≥ 2 times the current dose in SIRIUS and VENTURE, whereas any temporary increase in OCS dose during ZONDA and PONENTE was defined as an exacerbation. Nonetheless, a 32% reduction in exacerbations was seen in SIRIUS, compared with a 55–70% reduction in ZONDA and a 59% reduction in VENTURE with each respective biologic relative to placebo.

## Conclusions

Designs for OCS tapering studies have evolved over time. The design of the key single-center study [19] laid the foundations for improvements in study design that allowed OCS tapering methodologies to be successfully applied during larger, international, multicenter studies. Building upon the first steps taken by the key single-center study and SIRIUS, the duration and specificity of OCS tapering, as well as the monitoring of both disease control and safety during OCS tapering, have informed OCS-sparing asthma studies in recent years [19–24]. The studies to date show that stepwise OCS dose reduction under biologic therapy is possible while maintaining asthma control. While the protocols used during clinical trials are often complex, they provide important information on the effects of OCS-tapering methodologies that may be useful in clinical practice. Common features of these methodologies include a sufficient OCS dose optimization phase prior to initiation of biologic treatment, an end-target OCS dose tailored based on the OCS dose achieved during dose optimization, and careful monitoring for loss of asthma control and symptoms of adrenal insufficiency during tapering. Though adrenal insufficiency during OCS tapering remains a concern, the steps needed to reduce the risks are becoming clearer and better understood. Additionally, most schedules consisted of OCS dose reductions every 4 weeks, beginning with reductions of either 10 mg/day or 5 mg/day for patients with the highest baseline OCS doses and finishing with smaller reductions of 2.5 mg/day or 1.25 mg/day until the target dose was achieved. This general approach may be useful in real-world settings.

Future studies may identify genes that are up- or down-regulated during OCS tapering and these genetic markers as well as other biomarkers could help to provide a more patient-centered approach to OCS tapering, either prior to or during biologic therapy. Lessons learned in the development of effective OCS-tapering trial methodologies in asthma may be useful in the development of future trials in this area and will likely help guide OCS reduction in real-world patient care.

## Supplementary Information

**Additional file 1.** Criteria for not reducing oral corticosteroid (OCS) dose and definition for documented failures of OCS reduction within 6 months prior to enrollment (ZONDA).

## Data Availability

Data sharing is not applicable to this article as no datasets were generated.

## Abbreviations

ACQ: Asthma Control Questionnaire
AE: Adverse events
FEV_1_: Forced expiratory volume in 1 s
FVC: Forced vital capacity
HPA: Hypothalamic–pituitary–adrenal
ICS: Inhaled corticosteroid
ITT: Intent-to-treat
IV: Intravenous
LABA: Long-acting β_2_-agonist
OCS: Oral corticosteroids
PC20: Provocative concentration of methacholine resulting in a 20% decrease in FEV_1_
PD20: Provocative dose of methacholine resulting in a 20% decrease in FEV_1_
PEF: Peak expiratory flow
PRO: Patient-reported outcome
RCT: Randomized controlled trial
SC: Subcutaneous
