# Supplementary material for: Development of methodology for assessing steroid-tapering in clinical trials for biologics in asthma
Source: Respir Res. 2022 Mar 4;23:45. doi: 10.1186/s12931-022-01959-1 (PMC8896284; doi:10.1186/s12931-022-01959-1)
Supplement: Supplementary file 1 — Additional file 1. Criteria for not reducing oral corticosteroid (OCS) dose and definition for documented failures of OCS reduction within 6 months prior to enrollment (ZONDA). [file 12931_2022_1959_MOESM1_ESM.docx]

**Supplementary information**

**Development of methodology for assessing steroid-tapering in clinical trials for biologics in asthma**

Stephanie Korn, MD, Peter Howarth, MD, Steven G Smith, PhD, Robert G Price, MSc, Steven W Yancey, MSc, Charlene M Prazma, PhD, Elisabeth H Bel, MD

**Contents**

**Criteria for not reducing oral corticosteroid (OCS) dose**

**Definition for documented failures of OCS reduction within 6 months prior to enrollment (ZONDA)**

**Criteria for not reducing oral corticosteroid (OCS) dose**

The criteria for not reducing OCS dose for each study were as follows:

**Key single-center study:** exacerbation with an increase in the number of sputum eosinophils

**SIRIUS:** morning peak expiratory flow (PEF) <80% of baseline, mean asthma-related nighttime awakenings >50% increase over the baseline period, >4 rescue inhalations per day over baseline value for any 2 consecutive days or ≥12 inhalations in any day, ≥0.5-point worsening in Asthma Control Questionnaire-5 (ACQ-5) score, symptoms of adrenal insufficiency

**ZONDA:** pre-bronchodilator forced expiratory volume in 1 second (FEV_1_) <80% of baseline, morning PEF <80% of baseline, mean nighttime awakenings >50% increase compared with baseline, >4 short-acting β_2_-agonist rescue inhalations per day over baseline value or ≥12 inhalations per day, ≥0.5-point worsening in ACQ-5 score, increase in OCS dose for asthma symptoms

**VENTURE:** asthma symptom worsening, (≥0.5-point worsening in ACQ-5 score), clinically significant exacerbation, mean morning PEF <70% of baseline, pre-bronchodilator FEV_1_ <80% of baseline, >4 rescue inhalations per day over baseline value for any 2 consecutive days or ≥12 inhalations in any day, presence of adrenal insufficiency

**PONENTE:** asthma worsening or asthma exacerbation that requires increased OCS treatment (≥3 consecutive days), hospitalization or emergency room admission (patients who have a first asthma exacerbation or asthma deterioration will be allowed to continue OCS reduction after recovery on a slower schedule, based on the investigators’ judgment; in the case of a second asthma exacerbation or worsening, OCS tapering will cease). If any of the above criteria were met, patients continued their same OCS dose unchanged or treated the acute event (eg, exacerbation) and were then returned to the dosage one step higher than prescribed when the exacerbation occurred or criteria were met (see **Figure 1a** in main manuscript). Once receiving an OCS dose ≤5 mg/day, further reductions in OCS dose depended on cortisol concentration and adrenal insufficiency status, assessed by adrenocorticotropic hormone stimulation testing.

**Definition for documented failures of OCS reduction within 6 months prior to enrollment (ZONDA)**

Patients with documented failures of OCS dose reduction within 6 months prior to enrollment were not required to proceed through the dose optimization phase during run-in. Documented failures were defined as: a pre-bronchodilator FEV_1_ <80% of baseline, morning PEF <80% of baseline, nighttime awakenings increase of >50% of mean baseline, albuterol or salbutamol >4 puffs/day above mean baseline, requirement for OCS or OCS burst to treat an asthma exacerbation due to steroid reduction.
